# Ultrasensitive and Label-Free Detection of Phosphorylated Tau-217 Protein in Alzheimer’s Disease Using Carbon Nanotube Field-Effect Transistor (CNT-FET) Biosensor

**DOI:** 10.3390/bios15120784

**Published:** 2025-11-27

**Authors:** Jiao Wang, Keyu Yao, Jiahua Li, Duo Wai-Chi Wong, James Chung-Wai Cheung

**Affiliations:** 1 Department of Biomedical Engineering, Faculty of Engineering, The Hong Kong Polytechnic University, Hong Kong 999077, China; jiao.wang@connect.polyu.hk (J.W.); 23104323r@connect.polyu.hk (K.Y.); 2 Department of Clinical Laboratory, Hubei Provincial Hospital of Traditional Chinese Medicine, Wuhan 430073, China; 3 Shenzhen Research Institute, The Hong Kong Polytechnic University, Shenzhen 518057, China; koopa.l.li@connect.polyu.hk; 4 Research Institute for Smart Aging, The Hong Kong Polytechnic University, Hong Kong 999077, China

**Keywords:** p-tau217, CNT-FET, Alzheimer’s disease, biosensor

## Abstract

Early diagnosis of Alzheimer’s disease (AD) remains challenging due to the extremely low concentration of relevant biomarkers and the limited sensitivity of conventional detection techniques. In this study, we present a carbon nanotube field-effect transistor (CNT-FET) immunosensor for label-free detection of phosphorylated tau at threonine 217 (p-tau217). The device employs a Y_2_O_3_/HfO_2_ dielectric layer and gold nanoparticles (AuNPs) to improve biofunctionalization, with anti-p-tau217 antibodies immobilized on the CNT channels. In phosphate-buffered saline (PBS), the sensor exhibited a linear response over a concentration range of 3 fM to 30 pM (R^2^ = 0.973) and achieved a limit of detection (LOD) of 1.66 fM. The device demonstrated high selectivity, with a normalized signal response (NSR) for p-tau217 that was 5–6 times higher than for human serum albumin (HSA) and p-tau231, even at 1000-fold higher concentrations of these interferents. The sensor exhibited reproducibility with a relative standard deviation (RSD) of 4.8% (*n* = 9) and storage stability with only a 10% decrease in signal after 7 days at 4 °C. Mechanistic analysis indicated that the net positive charge and structural flexibility of the p-tau217 peptide led to a reduction in drain current upon binding, consistent with electrostatic gating effects in p-type CNT-FETs. Current limitations include the absence of standardized p-tau217 reference materials. Future work will focus on validation with clinical samples. This CNT-FET platform enables rapid, minimally invasive detection of p-tau217 and holds strong potential for integration into clinical workflows to facilitate early AD diagnosis.

## 1. Introduction

Alzheimer’s disease (AD), a progressive neurodegenerative disorder, is recognized as the predominant cause of dementia worldwide [1]. The World Health Organization (WHO) estimated that over 55 million individuals are affected by dementia, with AD contributing to 60–70% of these cases. A nearly threefold increase in global dementia prevalence by 2050 (139 million cases) will be attributed to aging populations, extended lifespans, and escalating comorbidities such as metabolic and cerebrovascular disorders [2,3,4]. A critical barrier in AD management lies in the delayed manifestation of clinical symptoms, which typically arise only after irreversible neuronal degeneration [5,6,7]. Despite a limited number of approved drugs for mitigating AD symptoms, a truly effective treatment remains elusive, underscoring our incomplete understanding of the disease. Consequently, combating AD currently relies heavily on early diagnosis, timely intervention, and symptomatic management [8,9]. Some cognitive training and physical exercise are also incorporated [10,11]. Early diagnosis is therefore critical, requiring sensitive and accessible biomarkers that can detect pathological changes before irreversible damage occurs. This has driven intensive research into blood-based biomarker quantification strategies that could enable routine screening in primary care settings.

The pathogenesis of AD is complex, featuring several markers associated with the etiology of the disease [12,13]. Established biomarker quantification strategies for AD, including β-amyloid (Aβ) and tau measurements in cerebrospinal fluid (CSF) or amyloid or tau positron emission photography (PET) imaging, have been of limited use for screening because of invasiveness, high costs, and the need for specialized personnel. Unlike CSF sampling, which requires invasive lumbar puncture, or PET imaging, which requires specialized facilities and radiopharmaceuticals, blood-based biomarkers generally have lower costs, are less invasive and have the potential to be deployed widely throughout the community, allowing for early and repeated testing [14,15,16]. Among these markers, phosphorylated tau proteins (p-tau) are the most promising blood biomarkers for AD research [17]. Tau has over 40 phosphorylation sites; however, little research has been carried out on their comparative diagnostic value, especially in plasma. Several studies demonstrated that p-tau protein isoforms, including p-tau181, p-tau217, and p-tau231, are highly specific for the detection of PET-confirmed Aβ and tau pathology across the clinical AD continuum [18]. A new isoform of p-tau at threonine 217 (p-tau217) has been identified with notable relevance in the pathological diagnosis of AD, indicating superior diagnostic utility compared to other biomarkers [19,20,21]. A recent study indicated that p-tau217 was the most important phosphorylation site in the differentiation between AD and control brain tissue, outperforming p-tau181 [22,23]. Unlike conventional biomarkers, p-tau217 levels in peripheral blood correlate strongly with cerebral amyloid-β (Aβ) alterations, detectable even prior to Aβ plaque deposition [19,21,24]. Longitudinal studies further establish p-tau217 as a unique indicator capable of tracking amyloid-driven pathological changes across both presymptomatic and symptomatic AD phases, positioning it as a dynamic monitor of disease progression. Its elevated concentrations in CSF and plasma further reinforce its diagnostic utility, driving innovations in minimally invasive detection methodologies [20,25,26].

Despite its promising diagnostic potential, the clinical translation of p-tau217 detection is hampered by several challenges. p-tau217 concentrations in plasma are approximately 10-fold lower than CSF levels in AD patients, necessitating detection methods with femtomolar sensitivity. The extremely low concentrations in blood samples demand detection methods far more sensitive than conventional approaches [27,28,29,30]. Furthermore, the substantial variability in blood composition complicates the accurate and reliable detection of blood-based biomarkers. Consequently, the development of an ultrasensitive detection method is crucial for effective plasma biomarker analysis [31,32,33]. Established techniques for biomarker detection—such as enzyme-linked immunosorbent assays (ELISAs), single-molecule array (Simoa) platforms, and mass spectrometry—are limited by their dependence on invasive CSF sampling, time-consuming procedures, and complex instrumentation [34,35,36,37]. Although blood-based assays reduce invasiveness, they face challenges such as ultralow biomarker concentrations (in the femtomolar to picomolar range) and significant matrix interference, which hinder their widespread use—particularly in resource-limited regions with a growing burden of Alzheimer’s disease [38,39]. In contrast, field-effect transistor (FET) biosensors represent a transformative approach, enabling label-free detection, fast response times, and scalable miniaturization by directly converting biomolecular interactions into measurable electrical signals [37,40,41,42].

Semiconductive carbon nanotubes (s-CNTs)—characterized by their nanoscale structure, exceptional charge carrier mobility, and good biocompatibility—have stood out as ideal channel materials for field-effect transistor (FET) designs [43,44,45]. Yet their high degree of sensitivity often undermines their ability to resist interference when used in complex biological samples, such as serum. Existing solutions, including floating-gate technologies, create a barrier between the channel and the sample to avoid direct contact; however, this approach extends charge-screening distances, which in turn lowers the sensor’s sensitivity [46,47]. Furthermore, the limited number of probe-binding sites on micro-sized interfaces restricts the linear detection range for target biomarkers [48,49]. Thus, striking a balance between sensitivity, anti-interference performance, and probe density remains a key prerequisite for advancing FET biosensors toward practical clinical use. CNT-FETs offer a viable solution to these hurdles by combining the distinctive properties of carbon nanotubes—outstanding electrical conductivity, atomic-level resolution, and high surface-to-volume ratios—with biorecognition components [45,50]. Pioneering studies validate CNT-FETs’ capacity for attomolar-level detection in diluted biofluids, outperforming conventional assays while streamlining sample preparation [45,51].

**Figure 1:**
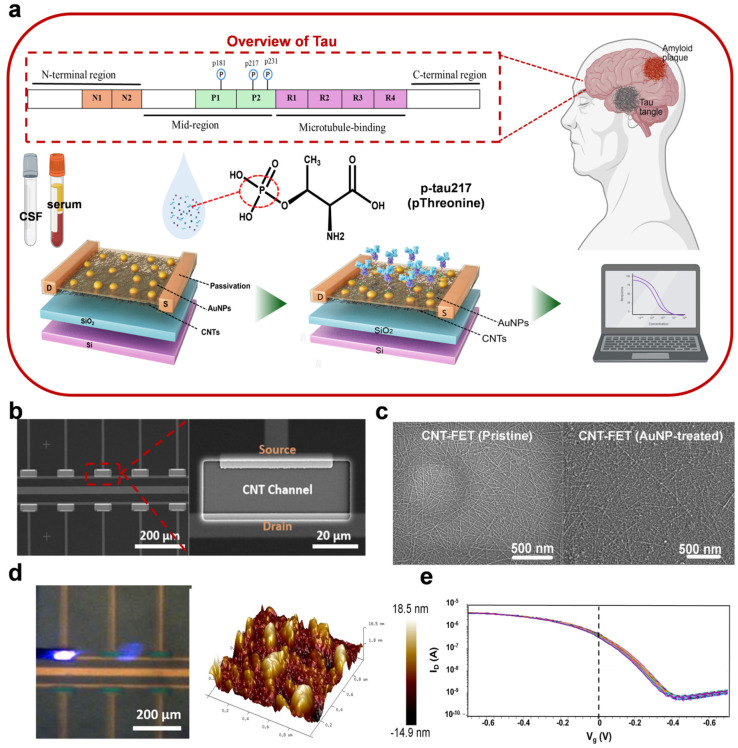
Fabrication and characterization of CNTFET biosensors. (**a**) Schematic illustration of the CNTFET biosensor for p-tau 217 detection; (**b**) SEM image of the CNTFET biosensor array; (**c**) SEM images of CNTFET biosensor chips on which AuNPs were uniformly distributed on the film; (**d**) AFM images of AuNP deposition; (**e**) Transfer curves of 50 CNTFETs (20 devices, 3 batches) after the deposition of Au nanoparticles, *V_ds_* = − 0.1 V.

The objective of this work was to develop a CNT-FET immunosensor for the ultrasensitive detection of phosphorylated tau-217 (p-tau217), as schematically illustrated in Figure 1a. To enhance biofunctionalization efficiency and ensure environmental stability, the CNT channels were modified with an ultrathin yttrium oxide (Y_2_O_3_) layer and a high-κ hafnium oxide (HfO_2_) dielectric layer, followed by decoration with gold nanoparticles (AuNPs) to enable monoclonal antibody (mAb) immobilization. This design provides straightforward fabrication, reliable functionalization, and label-free, real-time electrical detection using cost-effective digital measurement systems. Furthermore, the platform can be readily adapted for other clinically relevant biomarkers, such as p-tau231, underscoring its versatility in neurodegenerative disease diagnostics. The scope of this study includes the fabrication and functionalization of the CNT-FET device, systematic characterization of its sensing performance under varying concentrations of p-tau217, and evaluation of its sensitivity, selectivity, reproducibility, and stability for potential clinical applications.

## 2. Materials and Methods

### 2.1. Materials and Sample Preparation

Thioglycolic acid (TGA, >95% purity) was bought from TCI Co., Ltd. (Shanghai, China); N-hydroxysuccinimide (NHS, 98.5%), bovine serum albumin (BSA), and human serum albumin (HSA) were sourced from Sigma-Aldrich (St. Louis, MO, USA); 1-ethyl-3-(3-dimethylaminopropyl) carbodiimide hydrochloride (EDC, 98%) was purchased from Aladdin Biochemical Technology Co., Ltd. (Shanghai, China); and Tween-20 and nuclease-free phosphate-buffered saline (PBS) were purchased from Solarbio, Beijing, China. Other chemicals were obtained from ZuoKe Biotech Co., Ltd. (Shenzhen, China). The raw materials for the sensors were purchased from Hunan Yuanxin Sensing Technology Co., Ltd. (Changsha, China). Synthetic p-tau217 peptide (sequence: PGTPG SRSRT PSLPT(p) PPTREP KKVAV VRTPP) was provided by Chinese Peptide Bio. Co., (Shenzhen, China); the purity of the peptides was >98% as assessed by high-performance liquid chromatography (HPLC). The molecular mass (3344.8 g/mol) of the synthesized peptides was confirmed by Electrospray Ionization–Mass Spectrometry (ESI-MS). The corresponding P-tau217 antibody and p-tau231 peptide were purchased from Abcam (Cambridge, UK). The peptides were brought to room temperature before being dissolved in 1 mL of deionized water. PBS solution (10 mM, pH = 7.4) was used to serially dilute the stock solution into femtomolar to picomolar concentrations. A 1% (*w*/*v*) BSA solution and 0.05% Tween-20 in 1×PBS were freshly prepared for surface blocking. All additional chemicals and reagents were of analytical grade and utilized without further purification. Deionized water was obtained from the ELGA Purelab water purification system (18.2 MΩ.cm) (ELGA LabWater, UK).

### 2.2. Fabrication of CNT-FET Device

The fabrication of the CNT-FET sensor followed established protocols described in the previous literature [52,53]. First, polymer-sorted carbon nanotube solutions were cast onto 4-inch Si/SiO_2_ wafers. Next, source and drain electrodes—with a Ti/Pd/Au composition (thicknesses: 0.3 nm/40 nm/30 nm)—were patterned using electron-beam evaporation (EBE); subsequent reactive ion etching (RIE) was performed to delineate the channel regions. A yttrium (Y) film was deposited by EBE and subsequently thermally oxidized at 270 °C for 30 min to form an ultrathin yttrium oxide (Y_2_O_3_) layer (3 nm). A hafnium oxide (HfO_2_) dielectric layer (10 nm) was then grown by atomic layer deposition (ALD), after which AuNPs (2–4 nm) were added through magnetron sputtering to facilitate bio-probe conjugation. To ensure electrolyte isolation during device operation, the final step involved passivating the electrical contacts and interconnects with a photoresist (S1813) layer. Prior to undergoing functionalization and testing, all completed devices were stored in a vacuum desiccator.

### 2.3. Functionalization of CNT-FET Biosensors

The sensing interface of the floating-gate (FG) CNT-FET was functionalized through a multi-step protocol. Initially, the channel region was incubated with 0.5% (*v*/*v*) thioglycolic acid (TGA) at 4 °C for 12 h to facilitate Au-S covalent bond formation between TGA’s thiol groups and gold nanoparticles (AuNPs). Subsequently, carboxyl (–COOH) moieties on the TGA-modified surface were activated via a 1:1 mixture of 0.4 M 1-ethyl-3-(3-dimethylaminopropyl) carbodiimide (EDC) and 0.1 M N-hydroxysuccinimide (NHS), enabling covalent immobilization of anti-p-tau 217 monoclonal antibodies. For antibody immobilization, a 25 μL aliquot of 100 μg/mL antibody solution was dispensed onto the sensor’s sensing region and incubated at 4 °C overnight. Unbound antibodies were rigorously removed through sequential washing with phosphate-buffered saline (1×PBS, pH 7.4) and ultrapure water. To minimize non-specific adsorption, the sensor was incubated with 1% (*w*/*v*) BSA solution and 0.05% Tween-20 in PBS for 1 h at 25 °C, effectively blocking residual reactive sites. Finally, the functionalized device was rinsed with 1×PBS containing 0.05% Tween-20 and then ultrapure water, nitrogen-dried, and stored under inert conditions until use.

### 2.4. Far-UV CD Spectra Measurements

Far-UV circular dichroism (CD) spectra were acquired using a JASCO J-1500 Circular Dichroism Spectrometer (JASCO, Tokyo, Japan). For p-tau217 peptides, spectra were recorded over a wavelength range of 270 to 170 nm using a 0.1 cm rectangular quartz cuvette, with a step size of 1.0 nm. For the baseline correction, deionized water and PBS solution were used as blanks and automatically subtracted from the samples at the time of spectrum measurement. The sample measurement was collected as the average of three scans. The spectral data were collected at a scan rate of 50 nm min^−1^. Peptide samples were prepared at a concentration of 0.2 mg/mL in three different matrices: deionized water, 0.01×PBS, and 1×PBS, all adjusted to pH 7.4.

### 2.5. Electrical and Sensing Measurements

Electrical characterization of the CNT-FET devices was performed using a Keithley 4200A-SCS semiconductor parameter analyzer (Solon, OH, USA) integrated with a Cindbest CS-4 probe station (Shenzhen, China). For structural and elemental analysis, the devices were characterized via scanning electron microscopy (SEM) and energy-dispersive X-ray spectroscopy (EDX), both conducted using a Tescan CLARA system (Brno, Czech Republic). Furthermore, X-ray photoelectron spectroscopy (XPS) (Thermo Scientific Nexsa, Waltham, MA, USA) and Raman spectroscopy (Renishaw, Wotton-under-Edge, UK) were employed to further analyze changes in the elemental composition and chemical states of the sensor surface after modification. Atomic force microscopy (AFM) (Bruker Dimension edge, Germany) was employed to characterize the morphological changes in AuNPs deposition.

For p-tau217 immunodetection, the biosensor’s channel region was incubated with 25 μL target-specific human p-tau217 protein (at optimized concentrations: 3 fM, 30 fM, 300 fM, 3 pM, 30 pM) at 37 °C for 20 min. Following this, the devices were rinsed with PBS to eliminate unbound p-tau217, then with deionized water, and finally dried under a nitrogen stream.

During electrical measurements, a silver wire gate electrode was immersed in 0.01×PBS electrolyte and positioned perpendicularly above the channel to stabilize the applied gate potential (*V_g_*). The source-drain current (*I*_ds_) was recorded under a constant bias voltage (*V_ds_* = −0.1 V) while sweeping *V_g_* from −0.7 V to 0.7 V. Transfer curves were generated to establish the correlation between *I*_ds_ modulation and p-tau217 concentrations ranging from 3 fM to 30 pM. All measurements were conducted at a drain voltage (*V_d_*) of −0.1 V to minimize leakage currents and ensure signal fidelity. A schematic illustration of the CNT-FET-based biosensor for p-tau217 detection is provided in Figure 1a.

### 2.6. Data Analysis

Each experiment was performed in triplicate (*n* = 3), and standard deviations of current changes were represented by error bars in the corresponding graphs. Transfer curves were acquired by recording the source-drain current (*I_ds_*) using a probe station coupled to a semiconductor analyzer, with measurements performed under a sweeping electrolyte gate voltage (*V_g_*) and a fixed source-drain voltage (*V_ds_* = −0.1 V). To evaluate the sensor’s ability to translate antibody–antigen biorecognition interactions into quantitative signals, the change in electric current (Δ*I*) was monitored. This Δ*I* signal was calculated as the difference between the initial current output (*I*_0_) and the real-time current output (*I*). The response of a biosensor is typically defined as Response = (*I_ds_* − *I_ds_*_0_)/*I_ds_*_0_ at specific values of *V_ds_* and *V_gs_*, where *I_ds_*_0_ and *I_ds_* represent the drain current measured before and after the hybridization of the target protein, respectively. In this study, the sensor response was calculated within the linear operating regime of the CNT-FET, with *V_ds_* set to −0.1 V and *V_gs_* set to −0.6 V.

## 3. Results

### 3.1. Characterization and Fabrication of CNT-FET Biosensors

The fabrication and structural configuration of the CNT-FET immunosensor are depicted in Figure 1b. Source-drain (S-D) electrodes were fabricated via photolithography, electron-beam evaporation, and the lift-off technique. The sensor incorporates polymer-sorted carbon nanotubes (CNTs) with a semiconducting purity exceeding 99.99% as the channel material, Y_2_O_3_/HfO_2_ as the insulating layer, and gold nanoparticles (AuNPs) as TGA linker-functionalization sites for immobilizing monoclonal antibodies (mAbs) (Figure 1c). The CNTs form a well-aligned, densely packed network with uniform dispersion and robust inter-tube connectivity. This interconnectedness of CNTs facilitates enhanced electron transport through the network, thereby generating a robust electronic signal.

Transfer curves of 50 CNT-FETs were randomly measured from 20 devices across 3 batches (Figure 1e). Each batch consisted of 80 devices, fabricated and tested independently. All transistors exhibit p-type field-effect transistor (p-FET) characteristics, with a current on/off ratio of approximately 10^5^. This outcome attests to the high semiconducting purity of the sorted CNTs, which is crucial for ensuring the reliable electrical performance of the immunosensor.

### 3.2. Antibody Immobilization

**Figure 2:**
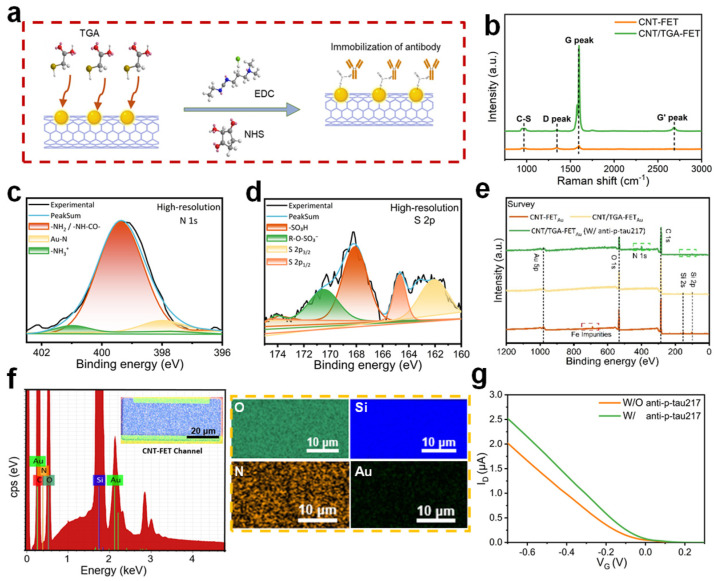
The CNTFET device was verified for antibody probe immobilization. (**a**) Schematic illustration depicting the procedure of immobilizing antibodies on the Au NPs across the CNT channel by employing Au NP functionalization with TGA linkers followed by EDCNHS crosslinking chemistry with the antibodies; (**b**) Raman spectrum of CNT before and after surface modification of TGA linker; (**c**–**e**) XPS spectra of N, S elements obtained after antibody decoration; (**f**) EDX shows that nitrogen exists on the surface of the channel; (**g**) transfer characteristic curves of the biosensor before and after antibody modification.

To comprehensively verify antibody immobilization, multiple complementary techniques were employed. SEM/AFM provided morphological evidence of surface modification, while XPS and EDS confirmed elemental composition changes consistent with protein attachment. Raman spectroscopy verified preservation of CNT electronic properties essential for sensor function. Figure 2a schematically depicts the procedure of antibody conjugation facilitated by multiple chemical reagents. To verify the successful immobilization of monoclonal antibodies (mAbs), complementary compositional analysis via energy-dispersive X-ray spectroscopy (EDS) detected nitrogen (N) signals within the channel region, corresponding to the nitrogen element in antibody residues (Figure 2f). Regarding X-ray photoelectron spectroscopy (XPS) analysis, as shown in Figure 2c–e, after incubation with the p-tau217 antibody, elemental peaks of N1s and S2p emerged, illustrating the successful immobilization of the antibody on the surface of gold nanoparticles (AuNPs). Raman spectroscopy (RS) is a potent technique for characterizing carbon-based materials and can effectively differentiate between pristine carbon nanotubes (CNTs) and CNTs modified with thioglycolic acid (TGA). All carbon-based materials show a G band around 1590 cm^−1^. Raman data confirms TGA successfully functionalizes CNTs without damage, keeping D and G bands while showing C-S bond features. CNT/TGA has a stronger G peak than pristine CNTs, suggesting TGA affects their graphitic order or electronics. Other peaks (like D and G’) also change slightly, indicating TGA alters CNT structure and vibrations (Figure 2b). These results ensure that the CNT-FET biosensor platform maintains excellent charge-transport capability while enabling specific antibody immobilization, which is fundamental for achieving high sensitivity and specificity in detecting target biomarkers like p-tau217.

The observed negative charge of the antibody is consistent with previous reports [37]. For the subsequent detection of p-tau217, an overnight incubation time of the p-tau217 antibody was utilized. The constructed sensor is a p-type device, which primarily conducts electricity through hole carriers. When large, negatively charged biomolecules (p-tau217 antibody) are immobilized or bound to the surface of the SWCNT channel, they introduce a local negative electrostatic potential in the vicinity of the nanotube. This negative surface potential exerts an electrostatic influence on the channel, effectively repelling electrons (minority carriers) and attracting holes (majority carriers) into the channel region. The accumulation of holes in the channel increases the carrier density available for conduction. Consequently, under a constant source-drain bias (*V_ds_*= −0.1 V), the source-drain current (*I*_ds_) through the p-type SWCNT channel increases due to the enhanced hole concentration. In summary, the p-tau217 antibody, which carries a substantial degree of negative charges, was immobilized onto the sensing interface and led to an induced electrostatic gating effect, resulting in an increase in the source-drain current (*I*_ds_) and a positive shift in the transfer curve at a given bias voltage. (Figure 2g)

### 3.3. Secondary Structural Stability Characterization of p-tau217 Peptides

**Figure 3:**
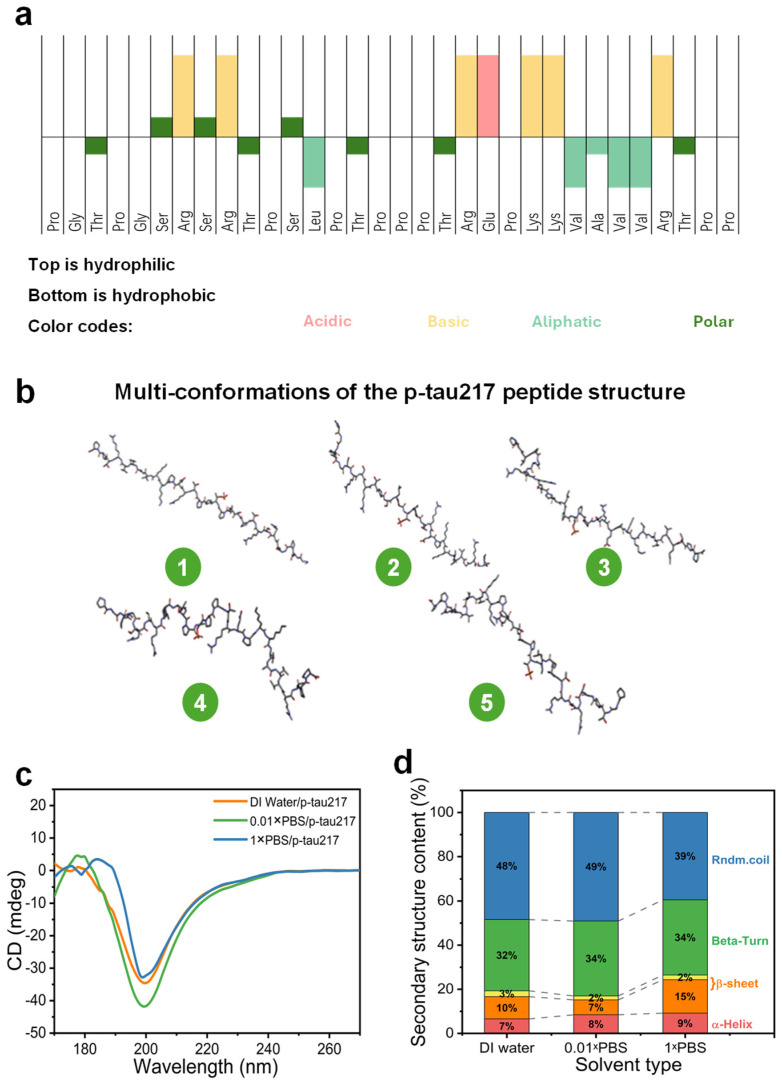
(**a**) Amino acid sequence of the p-tau217 peptide; (**b**) visualizing multiple conformations of the structures of p-tau217 peptide using software AlphaFold3 (five representative structures); (**c**) secondary structure of p-tau217 peptide in deionized water, 0.01×PBS, and 1×PBS; (**d**) quantitative analysis of secondary structure in solvents of different ionic strengths.

The properties of the p-tau217 polypeptide, obtained from the online platform (https://pepcalc.com/), provide crucial insights for related research (Figure 3a). The polypeptide consists of 31 residues, with a molecular weight of 3348 g/mol. Although phosphorylation introduces additional negative charge at specific amino acid residues, the overall polypeptide retains a net positive charge due to the predominance of basic amino acids within its sequence. At physiological pH, the phosphorylated peptide is likely to carry a net positive charge (+4) and have good water solubility. This pronounced positive charge promotes electrostatic repulsion with other cationic species and facilitates stable immobilization on negatively charged surfaces such as certain functionalized electrodes or oxide layers [54,55]. Moreover, the high content of proline (Pro) and charged residues supports structural flexibility and water solubility, which is advantageous for handling aqueous assay buffers [56]. From a biosensing perspective, these properties suggest that the peptide can be efficiently immobilized via electrostatic interactions, while its charge profile may significantly influence electrostatic gating responses in FET-based detection platforms.

We utilized AlphaFold3, the latest version of the AlphaFold protein structure prediction tool, to model the three-dimensional structures of our target peptides (Figure 3b). From the results, we selected five representative structure images for further analysis and visualization. All the images depict linear polypeptide chains with an extended conformation. Such a linear structure allows for the exposure of various functional groups (from amino acid side chains) along the chain, which is crucial for interactions like binding with antibodies [57]. Electrostatic attraction between the charged patches on the polypeptide and the oppositely charged regions of the antibody paratope facilitates the initial binding [58]. Additionally, the diverse side chains (indicated by the structural complexity in the images) can engage in other non-covalent interactions (such as hydrogen bonding and hydrophobic interactions) with the antibody, enhancing the specificity and affinity of the binding [58]. The linear conformation maximizes the contact area between the polypeptide and the antibody, promoting a stable antigen–antibody complex formation, which is a key aspect of its biological function and recognition processes [57].

The far-UV CD spectrum of p-tau217 peptides was recorded to investigate the secondary structure stability in deionized water, 0.01×PBS, and 1×PBS. As shown in the chart (Figure 3c), the secondary structure of the p-tau217 polypeptide in different solvents is mainly random coil and β-turn. The quantitative analysis of secondary structure, derived from circular dichroism spectral deconvolution, confirms that p-tau217 exhibits a predominantly disordered conformation characterized by high random coil (ranging from 39% to 49%) and β-turn (32% to 34%) content across different solvent environments, while structured elements such as α-helix and β-sheet constitute less than 10% and 15%, respectively (Figure 3d). The random coil and beta-turn content together indicate a largely disordered structure. The turn and coil regions exhibit exposed peripheral margins, rendering them highly susceptible to antibody binding. In contrast, the α-helical and β-sheet structures maintain a non-deformable conformation, which poses significant challenges to their integration with antibodies [59,60]. In the free state, the p-tau217 peptide predominantly adopts a random coil conformation and lacks stable secondary structures. Upon antibody binding, however, driven by non-covalent interactions (such as hydrogen bonding and hydrophobic interactions) from the complementarity-determining regions (CDRs) of the antibody, the polypeptide folds into an ordered structure required for antibody recognition—for instance, forming a β-turn or a short α-helix [61,62,63]. This conformational transition enables key moieties (e.g., the phosphorylated group at the 217th residue) to be precisely embedded into the specific binding pocket of the antibody, thereby achieving high-efficiency binding [62,64].

Understanding p-tau217 structural flexibility is crucial for interpreting the allosteric mechanism underlying sensor response. The predominantly disordered conformation (39–49% random coil) enables conformational changes upon antibody binding that directly influence electronic coupling with the CNT channel. The antibody can specifically bind to the peptides and reduce the possibility of non-specific binding.

### 3.4. Performance Verification of CNT-FET Immunosensor

**Figure 4:**
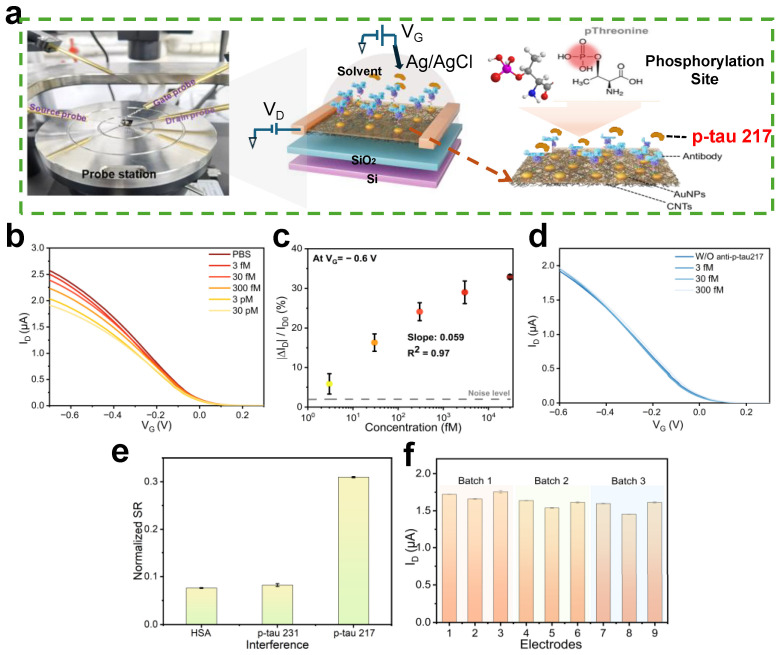
(**a**) Photo of the measurement setup and illustration of the CNT-FET biosensor for the p-tau217 detection; (**b**) the change in the transfer curve of the CNT-FET biosensor was recorded after the introduction of different p-Tau217 concentrations; (**c**) calibration curves at a series of p-tau217 concentrations (*n* = 3). The dashed line refers to threefold noise level; (**d**) transfer curve of a CNT-FET biosensor without modification of the antibody; (**e**) quantitative comparison of normalized SR (Δ*I*/*I*_0_) with the injection of target p-tau217 and non-target proteins. Error bars represent the standard deviation of three immunosensor replicates; (**f**) reproducibility of the constructed CNT-FET immunosensor.

Figure 4a illustrates the electrical connection setup of the device to a probe station, with clear labeling of the source (S), drain (D), and gate (G) terminals. To assess the sensitivity of the CNT-FET biosensor, antibody-functionalized chips were incubated with sequentially increasing concentrations of p-tau217, ranging from 3 fM to 30 pM. The current-voltage (*I*_ds_–*V_g_*) transfer characteristics were systematically recorded using a semiconductor parameter analyzer before and after each target incubation step, as shown in Figure 4b.

As shown in Figure 4b, the transfer curve exhibited a negative shift in gate voltage (*V*g) following the binding of p-tau217. When p-tau217 binds to the antibody, its net positive charge is brought into close proximity to the CNT channel. In a p-type CNT-FET, this local positive charge acts as an effective local gate, repelling holes and thereby reducing the channel conductance. This manifests as a decrease in the source-drain current (*I*_ds_) and a negative shift in the gate voltage (*V_g_*) required to achieve the same current, as observed in the transfer curve.

The negative shift in the transfer curve is due to an electrostatic gating effect, where the positively charged peptide modulates the local electrostatic environment at the CNT channel. This charge-based modulation is the fundamental mechanism by which the CNT-FET transduces the biochemical binding event into an electrical signal. The biosensor exhibits high sensitivity, as demonstrated by its linear response (*R*^2^ = 0.973) over a range of concentrations and an impressive limit of detection (LOD) of 1.66 fM, calculated on the linear regression equation (y = 0.064 + 0.059 lgC) and the 3-fold signal-to-noise ratio. This ultrasensitive detection capability underscores the effectiveness of the CNT-FET platform for biomarker analysis, relying on the precise interplay between antibody-mediated recognition and charge-induced modulation of the CNT channel conductance (Figure 4c). The specificity of this response is confirmed by the control experiment: in the absence of antibody functionalization, the CNT-FET shows no significant response to p-tau217, indicating that non-specific adsorption or direct interaction between the peptide and the CNT surface is negligible (Figure 4d). This highlights the crucial role of antibody–antigen interaction in mediating the sensor’s electrical response.

To evaluate the specificity of the sensor, human serum albumin (HSA) and p-tau231 (each at 25 µL, 10 μg/mL) were selected as interfering substances, as HSA is an abundant non-target protein in biological samples and p-tau231 is a homologous tau phosphorylation isoform that may potentially cross-react with the anti-p-tau217 antibody. These interfering substances were applied at relatively high concentrations (1000 times the typical concentration of p-tau217) to saturate any non-specific binding sites on the antibody or sensor surface, thereby maximizing the potential for non-specific responses and enabling a rigorous assessment of the sensor’s ability to discriminate between the target p-tau217 and interfering analytes. The sensor’s electrical responses to these high-concentration interferents were then recorded and compared with its response to the target p-tau217, with minimal signal deviation in the presence of HSA and p-tau231, indicating strong specificity of the sensor. From the data presented in Figure 4e, the biosensor exhibits distinct signal responses toward the target analyte and potential interferents, with notable differences in both concentration and normalized signal ratio (SR) values. When exposed to high concentrations of interferents—including 10 μg/mL human serum albumin (HSA) and 10 μg/mL p-tau231—the normalized SR values remain relatively low, at approximately 0.06 and 0.07, respectively. In striking contrast, even at a concentration 10,000-fold lower (10 ng/mL) than the interferents, the target p-tau217 elicits a significantly higher normalized SR of ~0.31. This differential response is particularly notable: the normalized SR for p-tau217 is approximately 5–6 times higher than that of the interferents, despite the target being present at a concentration three orders of magnitude lower. Such results strongly confirm the biosensor’s high specificity—even in the presence of structurally analogous peptides (e.g., p-tau231) and abundant serum proteins (e.g., HSA) at concentrations vastly exceeding that of the target. These results validate the robust specificity of the immobilized antibody and underscore the practical utility of the developed immunosensor for accurate biomarker detection in complex biological samples.

Reproducibility was assessed using nine sensors from each of three independently fabricated batches, all prepared under identical conditions. Each device was tested with 30 fM p-tau217 peptide. Error bars represent the standard deviation of three independent measurements at 30 fM p-tau217 for each of nine sensors. The measured responses showed a relative standard deviation (RSD) of 4.8% (at *V_g_* = −0.5 V), indicating high device-to-device reproducibility and batch-to-batch consistency (Figure 4f).

**Figure 5:**
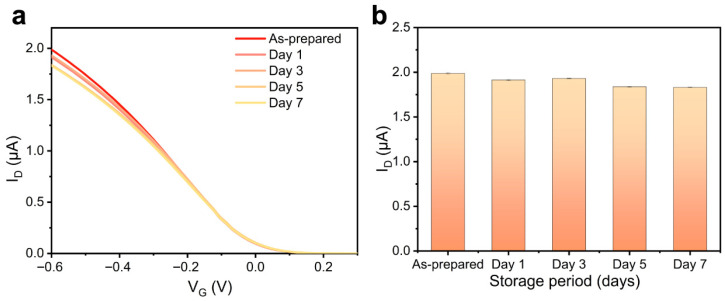
(**a**) Transfer curves of the antibody-modified CNT-FET biosensor and (**b**) drain current variation recorded after different periods of storage (*V_g_* = −0.6 V, *n* = 3).

The storage stability of CNT-FET immunosensors is a critical performance metric, influenced by factors such as device architecture, bottom-gate design, source-drain electrode materials, and the charge-transport properties of carbon nanotubes. To assess time-dependent stability, the fabricated immunosensor was stored at 4 °C following antibody functionalization, with its transfer characteristics periodically measured under consistent conditions. As shown in Figure 5a, representative transfer curves remain consistent across different storage durations. Quantitative analysis of the drain current over seven days (Figure 5b) reveals only a 10% decrease from the initial value, indicating minimal degradation. The slight, gradual decline in *I*_ds_ is attributable to repeated mechanical contact from probe needles during electrical measurements, which may cause minor electrode wear. Despite this operational artifact, the highly stable transfer characteristics confirm the robust structural and functional integrity of the biosensor over time, underscoring its suitability for practical applications requiring reliable long-term performance.

## 4. Discussion

This work demonstrates the first successful application of CNT-FET technology for p-tau217 detection, achieving femtomolar sensitivity suitable for blood-based AD diagnostics. While current measurements used benchtop instruments, future work will focus on integrating miniaturized, wireless electronics for portable point-of-care (POC) applications. This not only fills the technical gap between the high-sensitivity requirements of p-tau217 and portable diagnostic tools but also paves the way for the development of point-of-care (POC) devices for early AD screening—critical for initiating timely therapeutic interventions, as AD pathology often precedes clinical symptoms by decades. Current AD diagnosis relies heavily on cerebrospinal fluid (CSF) sampling (invasive) or neuroimaging (high-cost), limiting their accessibility in primary care settings. Blood-based p-tau217 detection, enabled by the ultra--sensitive CNT-FET platform herein, circumvents these barriers by leveraging minimally invasive sample collection and low-cost instrumentation. By demonstrating reliable detection of femtomolar p-tau217, the work validates the potential of CNT-FET technology to serve as a scalable, clinically viable tool for AD risk stratification and disease monitoring.

Beyond performance metrics, this study is the first to show how antibodies can be used to bind p-tau217 peptide and reveals fundamental insights into bioFET sensing mechanisms through the binding of antibodies and the conformation of peptides. The p-tau217 polypeptide exhibits a net positive charge and high water solubility due to its abundance of basic and proline residues, which facilitate stable immobilization on negatively charged biosensor surfaces and efficient handling in aqueous environments [56]. Structural predictions and spectroscopic analyses reveal that p-tau217 predominantly adopts a disordered, extended conformation in solution, exposing diverse functional groups that enable strong and specific interactions with antibodies through electrostatic attraction and non-covalent bonding [34]. Upon antibody binding, the peptide undergoes a conformational transition to more ordered structures, allowing precise positioning of the phosphorylated residue within the antibody’s binding pocket and enhancing recognition efficiency [61]. These combined physicochemical and structural properties make p-tau217 highly suitable for sensitive and selective detection in FET-based biosensing platforms.

Despite the above potential for clinical translation and novel mechanistic insights, this study has several limitations. First, the sensor’s performance was exclusively evaluated in PBS solutions that do not recapitulate the complexity of real biological matrices (e.g., human plasma or serum) targeted for clinical AD diagnostics. Second, the reproducibility and long-term stability of the CNT-FET sensor fabrication process and performance were not systematically characterized. Additionally, there are no certified quality control (QC) products for p-tau217 peptides on the market. This absence of standardized QC reagents has hindered the reliable comparison of detection results for p-tau217. The synthetic p-tau217 used in this study cannot fully mimic the structural and phosphorylational heterogeneity of endogenous tau in clinical samples.

This work represents proof-of-concept in idealized laboratory conditions. Clinical translation requires comprehensive validation in human plasma/serum matrices, addressing protein fouling, competitive binding, pH variations, and complex interferent mixtures. Future work will focus on two key directions. First, efforts will be dedicated to optimizing the sensor’s stability and anti-interference capability in complex biological matrices (e.g., plasma or serum), which contain abundant interfering substances such as human serum albumin, lipoproteins, and non-target tau isoforms that may induce non-specific adsorption on the carbon nanotube (CNT) surface or disrupt antibody-p-tau217 binding. Second, work will focus on developing a multi-biomarker CNT-FET sensor array for AD diagnosis. This integrated platform will involve functionalizing a CNT-FET array with highly specific antibodies targeting multiple AD-related biomarkers (e.g., p-tau217, p-tau231, p-tau181, Aβ1-42) and validating the platform using large-scale clinical cohorts to establish a comprehensive biomarker panel that supports early AD screening, disease staging, and monitoring of therapeutic responses.

## 5. Conclusions

In this study, we developed and validated a floating-gate carbon nanotube field-effect transistor (FG CNT-FET)-based immunosensor for the ultrasensitive and selective detection of p-tau217, a pivotal biomarker in AD pathogenesis. To the best of our knowledge, this represents the first application of CNT-FET technology for quantitative detection of p-tau217, addressing a critical gap in existing AD diagnostic strategies. The device exploits FET transduction principles, with anti-p-tau217 antibodies covalently immobilized on Au-S linker-modified CNT channels, bridging the source-drain electrodes. Specific antigen–antibody binding generated electrostatic gating effects that induced measurable modulations in source-drain current (Δ*I*_ds_), thereby enabling real-time, label-free detection.

Performance evaluations in phosphate-buffered saline confirmed a linear response across concentrations ranging from 3 fM to 30 pM (*R*^2^ = 0.97), with an ultralow detection limit of 1.66 fM—well within the physiological range of p-tau217 in blood and cerebrospinal fluid. The biosensor further demonstrated excellent specificity and stability, with antibody-coated devices retaining functionality and showing only ~10% signal variation after seven days of storage. Owing to its rapid turnaround time (<1 h), high sensitivity, and minimal sample requirements, this CNT-FET platform shows strong potential as a first-line screening tool for AD, with positive cases subsequently confirmed by standardized clinical assays. Integrating this sensing technology into clinical workflows could significantly accelerate early diagnosis and improve patient management in AD.

## Data Availability

Dataset available on request from the corresponding authors.
